# Genome Sequence of Polycyclovorans algicola Strain TG408, an Obligate Polycyclic Aromatic Hydrocarbon-Degrading Bacterium Associated with Marine Eukaryotic Phytoplankton

**DOI:** 10.1128/genomeA.00207-15

**Published:** 2015-03-26

**Authors:** Tony Gutierrez, Haydn F. Thompson, Angelina Angelova, William B. Whitman, Marcel Huntemann, Alex Copeland, Amy Chen, Nikos Kyrpides, Victor Markowitz, Krishnaveni Palaniappan, Natalia Ivanova, Natalia Mikhailova, Galina Ovchinnikova, Evan Andersen, Amrita Pati, Dimitrios Stamatis, T. B. K. Reddy, Chew Yee Ngan, Mansi Chovatia, Chris Daum, Nicole Shapiro, Michael N. Cantor, Tanja Woyke

**Affiliations:** a School of Life Sciences, Heriot-Watt University, Edinburgh, United Kingdom; b Department of Microbiology, University of Georgia, Athens, Georgia, USA; c DOE Joint Genome Institute, Walnut Creek, California, USA

## Abstract

Polycyclovorans algicola strain TG408 is a recently discovered bacterium associated with marine eukaryotic phytoplankton and exhibits the ability to utilize polycyclic aromatic hydrocarbons (PAHs) almost exclusively as sole sources of carbon and energy. Here, we present the genome sequence of this strain, which is 3,653,213 bp, with 3,477 genes and an average G+C content of 63.8%.

## GENOME ANNOUNCEMENT

*Polycyclovorans* algicola strain TG408 was isolated from a laboratory culture of the marine diatom Skeletonema costatum (CCAP 1077/1C) by enrichment with polycyclic aromatic hydrocarbons (PAHs) as the sole carbon source (1). The strain represents a novel genus of obligate hydrocarbonoclastic marine bacteria (OHCB) that exhibit a narrow nutritional spectrum, preferring to utilize aliphatic and aromatic hydrocarbon compounds and small organic acids (2). Notably, strain TG408 displays versatility for degrading two- and three-ring PAHs, consistent with the catabolic spectrum of members belonging to the obligate PAH-degrading genera Cycloclasticus (3) and Neptunomonas (4). Strain TG408 is a strictly aerobic and motile rod-shaped bacterium that is associated with various species of marine diatoms and dinoflagellates found in different seas and oceans worldwide (1) (our unpublished data).

Here, we report the genome sequence of P. algicola strain TG408. Genomic DNA was isolated, and the sequence was generated at the Department of Energy (DOE) Joint Genome Institute (JGI) using the Pacific Biosciences (PacBio) technology. A PacBio SMRTbell library was constructed and sequenced on the PacBio RS platform, which generated 262,160 filtered subreads totaling 814.1 Mbp. All general aspects of library construction and sequencing performed at the JGI can be found at http://www.jgi.doe.gov. The raw reads were assembled using HGAP (version 2.1.1) (5). The final draft assembly produced 1 scaffold containing 1 contig totaling 3.7 Mbp in size and with an input read coverage of 263.8×.

Genes were identified using Prodigal (6), followed by a round of manual curation using GenePRIMP (7) for finished genomes and draft genomes in <10 scaffolds. The predicted coding sequences (CDSs) were translated and used to search the National Center for Biotechnology Information (NCBI) nonredundant, UniProt, TIGRFam, Pfam, KEGG, COG, and InterPro databases. The tRNAscan-SE tool (8) was used to find tRNA genes, whereas rRNA genes were found by searches against models of the rRNA genes built from SILVA (9). Other noncoding RNAs, such as the RNA components of the protein secretion complex, and the RNase P were identified by searching the genome for the corresponding Rfam profiles using Infernal (http://infernal.janelia.org). Additional gene prediction analysis and manual functional annotation were performed within the Integrated Microbial Genomes (IMG) platform (http://img.jgi.doe.gov) developed by the Joint Genome Institute, Walnut Creek, CA, USA (10).

The complete genome sequence length was 3,653,213 bp, with a G+C content of 63.8%. The genome contains 3,477 genes (3,413 protein-coding genes), with function predictions for 2,818 of them. A total of 64 RNA genes were detected. Other genes that are characteristic for the genus are given in the IMG database (10).

### Nucleotide sequence accession number.

The draft genome sequence of P. algicola strain TG408 obtained in this study was deposited in GenBank as part of BioProject no. PRJNA224116, with individual genome sequences submitted as whole-genome shotgun projects under the accession no. JOMH00000000.
